# Community health service center-based cardiac rehabilitation in patients with coronary heart disease: a prospective study

**DOI:** 10.1186/s12913-017-2036-3

**Published:** 2017-02-11

**Authors:** Lixuan Zhang, Li Zhang, Jing Wang, Fang Ding, Suhua Zhang

**Affiliations:** Department of Geriatrics, the Third Hospital of Hebei Medical University, No 139 Ziqiang Road, Shijiazhuang, Hebei 050051 China

**Keywords:** Coronary Disease, Community Health Services, Exercise capacity, HRQoL, Rehabilitation

## Abstract

**Background:**

Despite considerable efforts to encourage participation, even in some developed countries, proportion of patients participating in institution-based cardiac rehabilitation (CR) programs remained sub-optimal. The present study was designed to investigate the acceptability of community health service center (CHSC)-based Cardiac Rehabilitation (CR), and examine its effectiveness in terms of changes in quality of life (QOL), psychological state and exercise capacity.

**Methods:**

A consecutive series of eligible patients was recruited from the health registration system of two CHSCs in Shijiazhuang, Hebei, China. Patients in intervention site were provided with CR (CR-group) while patients in non-intervention site were offered the usual care (UC-group). Data regarding health-related QOL (HRQoL), psychological state and exercise capacity (6-min walk test = 6MWT) were collected and compared at baseline and at 6 months post-intervention.

**Results:**

Among invited patients eligible for CR program, 65.3% participated, while 5.3% of the participants dropped out during follow-up. Patients in CR-group showed significant decrease in the scores for anxiety and depression as per the Hospital Anxiety and Depression Scale (HADS), along with marked increases in the Short-Form Health Survey (SF-12)-based Physical (PCS) and Mental Component Summary (MCS) scores. Moreover, the measurement of 6MWT showed a significant increase of 57.42 m walking distance among CR patients in contrast with a slight increase among UC patients.

**Conclusions:**

Given the high participation and low withdrawal along with considerable improvements in HRQoL, psychological state and exercise capacity, CHSC was likely to be the optimal setting for implementing CR for patients with CHD in China.

**Trial registration:**

ChiCTR-TRC-12002500. Registered 16 September 2012.

## Background

Despite the considerable advances in the treatment modalities and options, coronary heart disease (CHD) remains a major cause of morbidity and mortality worldwide, with an estimated 18 million deaths attributable to CHD annually [1]. In China alone, an estimated number of 20 million people are living with CHD [2] while annually more than 700 000 die of it, accounting for about 22.5% of all major causes of death [3]. With the rapid increase in prevalence and incidence of CHD, healthcare delivery models aimed at optimal secondary treatment and prevention have gained increasing attention around the world.

Randomized trials in recent decades [4], confirmed by meta-analysis [5], supported the role of cardiac rehabilitation (CR) in minimizing the risk and severity of CHD, improvement of functional capacity, enhancement of psychological well-being and reduction in the risk of further cardiac insults. However, the majority of these programs were primarily hospital-based (usually academic medical centers), where the implementation of the rehabilitation services were usually carried out by cardiologist or cardiac nurses [6] and the provisions were limited only to the supervising University or hospital settings. Unfortunately, in the context of limited health funding and associated scarcity of medical resources, it seemed difficult to provide consistent care to CHD patients depending solely on such hospitals [7]. Moreover, despite the considerable efforts to encourage participation, even in some developed countries, proportion of patients participating in these institution-based CR programs remained sub-optimal [8, 9], with the reported participation rate ranging between 9 and 29.5% [10, 11]. On the other hand, home-based CR as an alternative mode appeared to improve the participation in several programs, indicating that 96.1% of home participants received 5 contacts with a rehabilitation nurse whereas only 56.1% of centre-based participants attended this number of classes [12]. However, it did not reduce the healthcare costs substantially [13] and in terms of outcomes didn’t appear to be superior compared to the hospital-based programs [12].

In China, as a major model of primary care, community health service centers (CHSCs) play an increasingly important role in the prevention and control of non-communicable diseases [14]. All the CHSCs are operated and funded by the Government and they are responsible for providing necessary healthcare services to local residents. The range of services offered by CHSCs includes health education, family planning, immunization and rehabilitation. Management of serious chronic illnesses has been made mandatory by the Specifications of National Basic Public Health Services which was promulgated in 2007 and reinforced in 2009 [15]. Therefore, since then, timely registration, efficient treatment and adequate follow up are considered as the routine work of CHSCs. Given the multiple advantages of primary care in the management of chronic diseases as indicated by previous studies [16, 17], we conducted a CHSC-based CR program for CHD patients led by general practitioners and community nurses. Care delivery was through home visits on a personalized basis. The objectives of the present study were, 1) to investigate the acceptability of the CHSC-based CR programs in terms of participation and adherence rate, 2) to examine its potential positive influence on quality of life, psychological state and exercise capacity.

## Methods

### Study design and participants

Between January 2012 and January 2015, this study was conducted in the Yuhua district of Shijiazhuang, Hebei, China. For logistic benefit, proximity to the research institute was emphasized as an important factor for the site selection. Two community health centers, Yuxiang and Huaidi, which were 3 km apart from each other and adjacent to the research institute, were selected as the study sites. Approximately 150,000 residents were catered by each of these centers, located in the inner city area of Yuhua district. Both the centers had implemented the management system of chronic diseases in 1997, and were providing their range of services to CHD patients. Both centers had similar distribution of demographic and socioeconomic parameters and the standards of healthcare services provided to the local residents were also comparable. Yuxiang was randomized as the intervention site for the CHSC-based CR program while Huaidi were provided with usual care (UC) for chronic disease management and therefore served as the non-intervention (control site).

The sample size was calculated based on the assumption that the CR program would result in an increase of 56 m (SD = 100 m) in exercise capacity (determined by 6 min. walk test, 6MWT) and allowed for 10% loss to follow up [18, 19]. The target sample size of 132 participants (66 per group) would provide 90% power at 5% level of significance (two-sided) to show this difference. This sample size could be achieved during the study period with reference to the health registration system of CHSC, which showed that each center annually registered about 30 CHD patients.

During the aforementioned study period, CHD Patients were identified from the health registration system records of the CHSC. The list of the newly admitted patients was thoroughly searched by the research assistants, to prepare an exhaustive list of CHD patients registered in the center. Patients aged 30–75 years with a recent coronary event defined as acute myocardial infarction (MI), who had undergone percutaneous coronary intervention (PCI) or coronary artery bypass grafting (CABG) were eligible to be included as a subject. Patients were excluded if there were evidences of severe comorbidities, psychiatric illness or cognitive decline culminating into potential inability to fulfill the requirement of the current study. In addition, those who were not permanent residents or had already participated in hospital-based CR programs were also excluded from the study cohort.

After a detailed description of the study procedures, a written informed consent was obtained from each participant. The study content and procedures were reviewed and approved by the Institutional Ethics Review Board (IERB) of Hebei Medical University.

## Program implementation

### CR Group

A multidisciplinary research team comprising of dietitian, psychologist, physiotherapist, cardiologist and nursing staffs having expertise in CR was established in 2012. Responsibilities for designing the training modules, providing review and guidance to the program administrators, scheduling the follow-up plan and training of the community healthcare providers were specifically allocated to the individual experts of the team.

The first home visits for the patients registered in the intervention site were conducted by a team consisting of trained community physicians or nurses about 45 days after discharge from hospital. During this visit, a structured and detailed assessment was conducted to determine the clinical and psychological status of the patients. Based on the collected information, after a detailed discussion with the patient and seeking consultation from the program team, a community based exercise training plan was designed. The training schedule was individualized but followed the international recommendations, including 10–20 min warm-up, 20–40 min aerobic training plan according to their preferred training modality in their home environment, 10 min. cool down and 20 min. relaxation at a frequency of 6 days/week at an intensity of 11–13 (fairly light to somewhat hard on the Borg scale) [20]. Most recommended mode of exercise was walking at home or outside of the local surroundings, although participants were able to choose other modes (e.g. using facilities in community leisure centers) if preferred. Patients were taught to get familiarized with the training duration and were advised to exercise continuously throughout the session at the prescribed level of intensity during the following 3 months. Participants were also required to record their daily exercise sessions in respective log sheets designed for the study, in which individualized exercise prescriptions were provided detailing exercise duration, frequency and intensity. They were required to return the log sheets to their physicians monthly. Subjects with good compliance (defined by complete adherence to the prescription for at least 95% of days) were rewarded (electronic sphygmomanometers were presented to them). The primary caregivers of the participants were trained simultaneously and instructed to monitor the exercise process. Regarding safety issues when exercising, patients were asked to contact either the research staff or their general practitioner if they experienced any symptoms during or after exercising. Patients were advised not to increase exercise duration or intensity without the consent from their physicians.

All patients and their caregivers were offered counseling, in which the topics of simple diet control with low fat, low salt and less sweets, stress reduction along with, if required, smoking cessation were covered. After the first visit, three additional visits were planed, at intervals of 3 weeks, 2 months and 6 months, respectively, with required adjustments and optimal encouragement for the maintenance of the training plan. In between the visits, 2–3 phone calls were made at regular intervals by community physicians or nurses to resolve issues, if any. In addition, they were encouraged to exchange their contact information and organize group discussion to share their experiences on exercise training, stress management, lifestyle modification among themselves. Two trained community nurses were designated to promote the group activities and responsible for providing consultation for them during the whole period of the study.

### UC Group

Patients in the non-intervention site (B) received usual care for the routine management of chronic diseases by community physicians and nurses. Alike the CR group, they were also provided with risk factor intervention and medication consultation through telephone calls or community visits. However, very few patients could avail or afford CR, as the CR delivery system was quite underdeveloped in China and not covered by basic medical insurance [21].

### Outcome measurements and data collection

Demographic, clinical and behavioral data were collected at baseline by interviewing patients and/or reviewing their medical records. Medication adherence was calculated as the percent of patients having 80% of days covered for medications prescribed by physicians. Outcome variables including health-related quality of life (HRQoL), anxiety and depression, exercise capacity (determined by 6MWT) and behavior or clinical risk factors were collected at baseline and at 6 months after intervention (follow up).

The 12-Item Short-Form Health Survey (SF-12v2) was used to measure HRQoL. SF-12 was the shorter health self-administered questionnaire derived from the SF-36. Two subscales were derived from the SF-12: the Physical Component Summary (PCS), an index of overall physical functioning and the Mental Component Summary (MCS) which was an index of emotional and mental health. The PCS and MCS were standardized to a mean of about 50, with higher scores indicating better self-perceived health. SF-12 was previously validated for the measurement of HRQoL among Chinese [22].

Anxiety and depression were estimated by Hospital Anxiety and Depression Scale (HADS) [23]. The HADS was a 14-item self-report questionnaire, measuring anxiety and depression through 7 items rated on a 4-point likert-type scale, respectively. Total scores ranged from 0 to 21. Higher score indicated affective symptomatology.

The assessment was conducted at the research institute as already arranged. Method for measurement was standardized through pre-investigation. Specific training on the guideline and skills for measurement and testing was conducted among investigators responsible for data collection. Investigators were blinded to which group the participants were assigned.

### Statistical analysis

Statistical analysis was performed using SAS statistical software version 8.2. Only patients who completed both baseline and follow up assessments (6 months post-intervention) were included in the final analysis. Variables were described by frequencies and percentages for categorical variables and mean ± standard deviation (SD) for continuous variables. Differences between the 2 study cohorts were compared using chi-square analysis for categorical variables and Student-*t* test for normally distributed continuous data.

## Results

In the intervention CHSC, a total of 95 patients eligible for CR were consecutively identified from the new admission register. Among these patients, 62 (65.3%) agreed to participate in the CHSC-based CR program. During the 6-months follow up, 5 (5.3%) withdrew themselves from the program. The reasons for withdrawal were enquired and it was found that, 4 subjects did not want additional individuals to get involved in their care and 1 moved to hospital-based CR program. During the 3-months’ exercise training, there were 72 (75.8%) subjects fully complying with all the recommendations and 48 (84.2%), 52 (91.2%) and 47 (82.5%) complying with the prescribed exercise duration, frequency and intensity, respectively. In the non-intervention CHSC, 69 out of 91 patients eligible for CR accepted assessment at enrollment and 6 months thereafter. No patients received hospital-based CR program during the follow up.

Demographic and clinical characteristics of the patients were presented in Table 1. The mean age was 63.7 years and 29.5% were women. Compared to patients on UC, the patients on CR were older, had higher proportion of women, retired and less educated. In addition, CHD patients having concomitant diabetes, hypertension and peripheral arterial disease were more among the subjects in the intervention (CR) arm as compared to the non-intervention (UC) arm, while the scenario was reverse for the patients with COPD. There were no significant differences across groups in terms of income, medical diagnosis and management strategies.

**Table 1:** Distribution of the CHD patient characteristics across the intervention groups (*n* = 146)

Characteristics	Total (*n* = 126)	CR group (*n* = 57)	UC group (*n* = 69)	*p*
Age, years	63.1 ± 7.3	64.7 ± 7.2	59.8 ± 7.6	<0.01
Gender, Female	30(29.5)	23(41.2)	7(10.1)	<0.01
Married	124(97.8)	56(97.6)	68(98.2)	0.84
Education: Junior high school or lower	58(47.9)	31(54.1)	27(39.2)	0.03
Retired	77(65.8)	44(77.4)	33(47.8)	<0.01
Income: (Chinese Yuan/month)				0.90
Low	42(32.9)	18(31.5)	24(34.3)	
Median	73(58.2)	34(59.6)	39(57.2)	
high	11(8.9)	5(8.9)	6(8.5)	
Medical diagnosis				0.92
STEMI	57(46.2)	27(47.4)	30(44.2)	
NSTEMI	43(34.1)	19(33.4)	24(35.1)	
Unstable angina	25(19.7)	11(19.2)	14(20.7)	
Management strategies				0.57
PCI	120(95.9)	55(96.6)	65(94.7)	
CABG	6(4.1)	2(3.4)	4(5.3)	
Co-morbidity				
COPD	33(28.6)	19(33.7)	14(20.3)	0.10
Diabetes	36(30.8)	22(38.2)	14(20.3)	0.02
Hypertension	55(48.6)	33(58.2)	23(33.3)	<0.01
PAD	26(22.6)	17(29.2)	9(13.1)	0.02

*CR* cardiac rehabilitation, *UC* usual care, *PCI* percutaneous coronary intervention, *STEMI* ST segment elevated myocardial infarction, *NSTEMI* non-ST segment elevated myocardial infarction, *CABG* coronary artery bypass graft, *COPD* chronic obstructive pulmonary disease, *PAD* peripheral arterial disease

As shown in Table 2, patients in two groups at baseline had similar HADS depression score, SF-12 PCS score, measures of 6MWT, weight, BMI, and equivalent distribution of some modifying factors including smoking and adherence to medication. However, the HADS anxiety score was significantly higher in CR patients than UC patients, while the SF-12 MCS score was obviously lower among CR patients than UC patients.

**Table 2:** Baseline characteristic of modifying risk factors across the intervention groups

	CR group (*n* = 57)	Usual care group (*n* = 69)	*p*
Anxiety and depression			
HADS anxiety score	8.39 ± 3.15	8.18 ± 3.42	0.03
HADS depression score	7.43 ± 3.22	7.07 ± 3.16	0.21
SF-12			
SF-12 PCS	38.7 ± 8.5	40.8 ± 8.2	0.14
SF-12 MCS	40.6 ± 9.7	44.5 ± 9.1	0.02
Exercise capacity			
6MWT, m	501.2 ± 73.4	511.8 ± 80.4	0.41
Modifying risk factors			
Medication adherence	28 (49.4)	35 (50.9)	0.82
Current smoker, n, %	39 (68.5)	45 (64.9)	0.65
weight	72.4 ± 16.5	71.6 ± 15.8	0.77
BMI	26.2 ± 4.2	25.9 ± 3.7	0.66

*HADS* hospital anxiety and depression scale, *MCS* mental component summary scale of SF-12, *PCS* physical component summary scale of SF-12, *6MWT* 6 min walk test, *BMI* body mass index

As presented in Table 3, the HADS anxiety score and HADS depression score displayed a marked decrease while the SF-12 PCS and SF-12 MCS scores showed an obvious increase at 6 months in comparison with the baseline value, implying an improved psychological status and better quality of life favoring CR. Although a similar decrease in HADS anxiety score and HADS depression score as well as a slight increase in SF-12 MCS score were also observed in UC patients at 6 month, the differences were not significant. Moreover, the measurement of 6MWT showed a significant increase of 57.42 m in CR patients from baseline to 6 months follow-up, while a slight decrease was observed among UC patients. Additionally, the proportion of the modifying factors like smoking and adherence to medication showed an obvious decrease during follow up in both groups. Body weight and BMI also decreased in both groups. However, the differences were not significant.

**Table 3:** Changes in the behavior and clinical risk factors at 6-months follow up (compared to baseline)

	CR group (*n* = 57)		Usual care group (*n* = 69)		*P* ^a^
	change	95% CI	change	95% CI	
Anxiety and depression					
HADS anxiety score	−0.69	−1.34, −0.46	−0.31	−1.18, 0.56	<0.01
HADS depression score	−1.54	−2.04, −1.04	−0.54	−1.27, 0.19	0.03
SF-12					
SF-12 PCS	8.70	6.05, 11.34	−3.4	−5.72, −1.08	<0.01
SF-12 MCS	11.50	8.02, 14.98	1.9	−0.44, 4.24	<0.01
Exercise capacity					
6MWT, m	57.42	41.60, 73.20	−9.8	−33.60, 14.00	<0.01
Modifying factors					
Medication adherence	−23.2%	−37.3%, −14.1%	−17.6%	−25.3%, −7.7%,	0.71
Current smoker, n, %	−12.6%	−18.5%, −5.9%	−9.7%	−12.0%, −2.3%	0.51
weight	−1.6	−5.0, 1.8	−0.5	−4.8, 3.8	0.82
BMI	−0.4	−3.8, 3.0	−0.2	−1.3, 0.9	0.88

*HADS* hospital anxiety and depression scale, *MCS* mental component summary scale of SF-12, *PCS* physical component summary scale of SF-12, *6MWT* 6 min walk test, *BMI* body mass index

^a^comparison between groups at 6 months

## Discussion

In this study, we investigated the effectiveness of CR program in the setting of CHSC. Among the eligible patients, 65.3% agreed to participate in the CHSC-based program and only 5.3% did withdraw themselves during follow up. CR patients displayed important improvement in the psychological and physical status between baseline and 6-months follow-up, including a significant decrease in HADS anxiety score and HADS depression score, marked increase in SF-12 PCS and SF-12 MCS scores as well as in exercise capacity as measured by 6MWT. These findings suggested that incorporation of CHSC-based delivery of CR program in the management of CHD patients was probably feasible.

Although the benefits of CR had been well established in patients with CHD, the reported proportions of participation in previous hospital or center-based CR programs were far less than optimal. According to data from the European Cardiac Rehabilitation Inventory Survey [24], fewer than half of eligible cardiovascular patients benefitted from CR in most European countries. In the United States, a health survey by the Behavioral Risk Factor Surveillance System (BRFSS) indicated that less than one third of eligible patients participated in CR programs [25]. Similarly, in a retrospective cohort study in Australia [26], Scott et al. reported that only 29% were referred to an outpatient cardiac rehabilitation (OCR) program, and fewer than a third of all referred patients actually attended the OCR programs. Compared to these reports regarding participation in hospital or center-based CR programs, our data showed a much higher proportion of participation (62.3%) and lower drop out (5.3%), suggesting that CR program might be more attractive to CHD patients in the setting of CHSC compared to hospitals. These perceived benefits of CHSC-based CR programs might include some well-known advantages of community-based programs like the close proximity, perceived convenience and easy access, as indicated by the previous studies [14, 27] and they could possibly explain our results to some extent. Potential explanations might also include the fact that some disadvantaged subgroups such as older patients, those having multiple-complications or with lower educations were more likely to prefer the CHSC-based programs as opposed to the hospital-based ones for simple logistic reasons.

Psychological intervention, as indicated previously [28], was pivotal for any successful rehabilitation programs, as the burden of psychological problems like anxiety and depression, were found to be considerably high among CHD patients [29], and these problems were found to be negatively associated with motivation, adherence, maintenance and prognosis [29]. A variety of psychological intervention techniques, including stress management [30], relaxation and meditation practices [31], were applied in previous CR programs and shown favorable effects in terms of reduction in anxiety and depression scores as well as HRQoL score. In the present study, psychological intervention was also one of the key components of rehabilitation program and might be the explanation for the substantial changes of HADS anxiety and depression scores as well as SF-12 PCS and SF-12 MCS scores. However, given the study setting, we anticipated, some further reasons underlying the results might also be important and therefore were needed to be pointed out. In this CHSC-based CR program, patients could receive education and guidance from the same team members who were easily available locally at the time of requirement. Moreover, the multidisciplinary management team could bridge the gap between primary and tertiary care thus minimizing the worries of the patients regarding availability of treatment at the time of emergency. The appreciation of these advantages could have helped the patients to gain confidence on health service providers, to participate in CHSC based programs actively and thus to enhance their health related quality of life.

Previous studies consistently confirmed the role of CR in improving the exercise capability of CHD patients [4, 32]. Results from the current study also showed a significant increase of 57.42 m in the distance walked by the patients participating in the CR program, suggesting that the CHSC-based CR program could also improve the capacity of physical exercise among the CHD patients. In addition, the results revealed a marked reduction of several modifying risk factors like smoking and improvements in adherence to medication among patients in both groups. However, there was no significant difference between two groups at 6 month regarding these. Potential explanations might include the fact that CHD patients, as the core population for chronic disease management in CHSC, were well educated on these issues and thus the potential for differences in these factors diminished across the groups.

In the present study, patients were recruited and allocated in a non-random way. The potential healthy volunteer effect might have resulted in an overestimation of the actual effects of CHSC-based CR program. Although randomized approach would have been ideal, it was not used in this study, considering the difficulty of randomization of patients in the setting of CHSCs. The patients, practitioners and community nurses were residing in the same community for years. Prior acquaintance and familiarity would result in inevitable crossing over between study arms through regular communication and mutual learning between participants and nonparticipants about skills on CR, which in all possibilities would have reduced the exposure contrast between the study groups. On the other hand, by virtue of recruiting all intervention recipients from the same CHSC, it was easier for the patients and their caregivers to get involved in mutual communication and group discussion. These events cumulatively facilitated better delivery of information regarding potential benefits of CR and enhanced their motivation to participate, which was just what we expected and might well be considered as one of the main advantages of CHSC-based CR programs. Unlike other studies, we did not set a hospital-based CR program as the control for comparison. So it remained unclear whether the present program could achieve benefits equivalent or more than the hospital-based CR program. However, in China, owing to the lack of prioritization and consequent underinvestment, CR services were mainly provided by university-based centers or a few private hospitals. Patients recruited in these programs were highly selective and inappropriate to be served as the control. [33]. But we expect, with passage of time, as CR programs will become more common, future studies will have the opportunity to provide further evidences regarding the efficacy of CHSC-based CR program with better comparison.

## Conclusions

In the present study, we investigated the effects of CR program for CHD patients in the setting of CHSC. Data indicated an optimal participation rate (65.2%) among eligible patients for CR and low withdrawal rate (5.3%) among participants. Moreover, patients completing the CHSC-based CR program displayed a substantial improvement in HRQoL, exercise capability and psychological state as comparison to UC patients. Findings from the present study suggested that CHSC was likely to be the optimal setting for implementing CR for patients with CHD in China.

## Abbreviations

6MWT: 6-min walk test
CHSC: Community health service center
CR: Cardiac rehabilitation
HADS: Hospital anxiety and depression scale
HRQoL: Health-related QOL
MCS: Mental component summary
QOL: Quality of life
SF-12: The short-form health survey
